# Therapeutic Potential of Secreted Amyloid Precursor Protein APPsα

**DOI:** 10.3389/fnmol.2017.00030

**Published:** 2017-02-07

**Authors:** Bruce G. Mockett, Max Richter, Wickliffe C. Abraham, Ulrike C. Müller

**Affiliations:** ^1^Department of Psychology, Brain Health Research Centre, Brain Research New Zealand, University of OtagoOtago, New Zealand; ^2^Department of Functional Genomics, Institute for Pharmacy and Molecular Biotechnology, Heidelberg UniversityHeidelberg, Germany

**Keywords:** Alzheimer’s disease, amyloid precursor protein, APPsα, synaptic plasticity, neuroprotection

## Abstract

Cleavage of the amyloid precursor protein (APP) by α-secretase generates an extracellularly released fragment termed secreted APP-alpha (APPsα). Not only is this process of interest due to the cleavage of APP within the amyloid-beta sequence, but APPsα itself has many physiological properties that suggest its great potential as a therapeutic target. For example, APPsα is neurotrophic, neuroprotective, neurogenic, a stimulator of protein synthesis and gene expression, and enhances long-term potentiation (LTP) and memory. While most early studies have been conducted *in vitro*, effectiveness in animal models is now being confirmed. These studies have revealed that either upregulating α-secretase activity, acutely administering APPsα or chronic delivery of APPsα via a gene therapy approach can effectively treat mouse models of Alzheimer’s disease (AD) and other disorders such as traumatic head injury. Together these findings suggest the need for intensifying research efforts to harness the therapeutic potential of this multifunctional protein.

## Introduction

**Figure 1:**
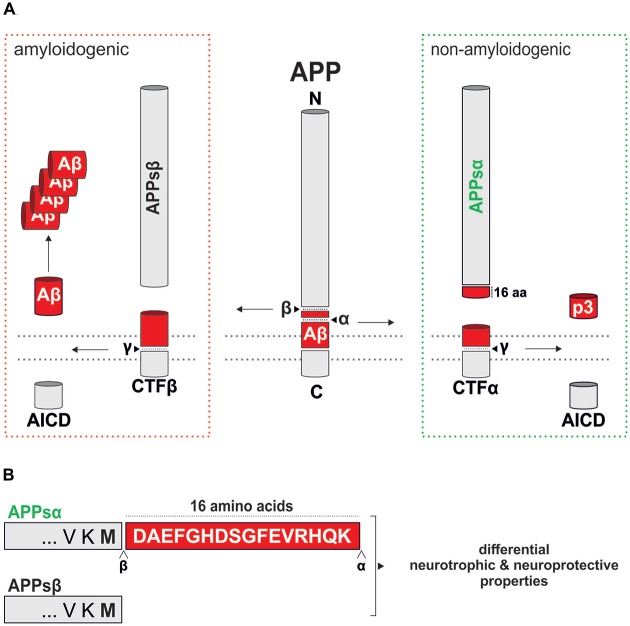
**Proteolytic processing of murine amyloid precursor protein (APP). (A)** Schematic overview of murine APP processing via the amyloidogenic (red dotted box) and the non-amyloidogenic (green dotted box) pathways. Processing by α-secretase within the amyloid-β (Aβ) region prevents Aβ generation and leads to the secretion of secreted APPα (APPsα) and harmless p3. In contrast, processing along the amyloidogenic pathway by β- and γ-secretase cleavage leads to Aβ production and liberates APPsβ. An intracellular fragment (AICD) involved in transcriptional regulation is released in both pathways. The position of cleavage sites is indicated by arrowheads. **(B)** Comparison of the C-termini of APPsα and APPsβ that differ only in the last 16 residues (highlighted as a red box).

Secreted amyloid precursor protein-alpha (APPsα, also known as soluble APPα), when generated from the neuronally expressed APP695 isoform by the action of α-secretase (Figure 1), is a 612 amino acid protein that was first shown in the mid-1990s to promote the survival and growth of cultured neurons under physiological and non-physiological conditions (e.g., glucose and oxygen deprivation, amyloid-β (Aβ) toxicity; Mattson et al., 1993; Barger and Mattson, 1996a; Furukawa et al., 1996). These observations have been supported and extended by myriad reports over the intervening years (Ryan et al., 2013; Hefter et al., 2016) and has generated suggestions that the promotion of α-secretase cleavage of APP and increasing APPsα levels could be a therapeutic strategy for the treatment of Alzheimer’s disease (AD; Turner et al., 2003; Ring et al., 2007; Postina, 2012; Hick et al., 2015; Fol et al., 2016; Habib et al., 2016) and possibly other neurological disorders. The purpose of this review is to consider the extent to which APPsα generation may be disrupted in AD, and summarize the many positive functions of APPsα that could be lost in the disease. In addition we will discuss the potential that either enhancement of non-amyloidogenic processing of APP or upregulating the expression of APPsα by other means has for preventing or at least slowing the progression of AD as well as treating other neurological disorders.

## APP Processing

APP is a single pass type I transmembrane protein that undergoes complex proteolytical processing by several enzymes termed secretases. In the amyloidogenic pathway, APP processing is initiated by β-secretase (β-amyloid cleaving enzyme, BACE-1), a transmembrane aspartate-type protease (for review see Vassar et al., 2014) that cleaves APP at the N-terminus of Aβ, leading to the secretion of the soluble ectodomain APPsβ (Figure 1A). In the competing and physiologically predominant non-amyloidogenic pathway, α-secretase cleaves APP within the Aβ region (Figure 1A), in a process that can be stimulated by neuronal and synaptic activity (Hoey et al., 2009; Hoe et al., 2012). This not only prevents the formation of Aβ peptides but also leads to the secretion of the ectodomain APPsα, which is only 16 amino acids longer than APPsβ (Figure 1B), into the extracellular space. Several members of the ADAM (a disintegrin and metalloprotease) family including ADAM9, ADAM10 and ADAM17, transmembrane Zn-proteases located at the cell surface, are able to cleave APP at the α-secretase site *in vitro* (reviewed by Saftig and Lichtenthaler, 2015). In neurons ADAM10 serves as the major physiological α-secretase as demonstrated by pharmacological inhibition and knockdown *in vitro*, as well as brain-specific knockout (KO) *in vivo* (Kuhn et al., 2010; Colombo et al., 2013; Prox et al., 2013). Subsequent processing of the membrane tethered C-terminal fragment resulting from β-secretase activity (CTFβ) by γ-secretase liberates Aβ and the APP intracellular domain (AICD), while CTFα processing yields the p3 fragment. γ-secretase cleavage occurs within the membrane by a complex of transmembrane proteins containing as a catalytic core presenilin (PS) 1 or 2. In wild-type neurons the predominant Aβ species generated is Aβ40, whereas familial forms of AD (FAD) linked to PS1 mutations result in a higher proportion of longer, more aggregation prone Aβ species including Aβ42 and Aβ43 that are believed to trigger plaque deposition (Veugelen et al., 2016).

## Alzheimer’s Disease

AD is a progressive neurodegenerative disease for which aging is the most significant risk factor. It has traditionally been diagnosed by the appearance of functional deficits that frequently begin with self-reporting of impaired episodic memory (Dubois et al., 2007). Definitive diagnosis, however, requires post-mortem confirmation, although in recent times a number of biomarkers are providing new ways of diagnosing in life, such as medial temporal lobe atrophy with hippocampi volume loss, abnormal cerebrospinal fluid levels of the neurotoxic Aβ peptide and tau protein, plus positron emission tomography evidence for amyloid plaques and reduced glucose metabolism (Jack and Holtzman, 2013). While the proximal causes of sporadic AD are largely unknown, the familial forms arise when any one of several autosomal dominant mutations in genes regulating the production and clearance of Aβ are present (Dubois et al., 2007, 2010).

The post-mortem neuropathology of AD is characterized by the extensive development of extracellular plaques containing Aβ that are generated by amyloidogenic processing of APP (Figure 1A), intraneuronal hyperphosphorylated tau leading to neurofibrillary tangles, neuroinflammation and cell loss. Moreover, accumulation of intraneuronal Aβ has been observed as an early event in transgenic animal models (Kumar et al., 2013) and may contribute to pathogenesis (Zou et al., 2015; Ji et al., 2016). Sub-clinical progression of AD may occur over 15–20 years prior to diagnosis (Jack and Holtzman, 2013). This early phase of the disease is characterized by the formation of soluble oligomeric forms of Aβ that cause neuronal dysfunction and toxicity that may underpin early cognitive deficits. At the center of this early dysfunction in particular is impairment of synaptic function. Investigations in both AD patients and in mouse models of AD have revealed significant reductions in dendritic spine density in both cortical and subcortical regions early in the disease that are highly correlated with the appearance of cognitive deficits (Scheff et al., 1990, 2006; Terry et al., 1991; Spires-Jones and Knafo, 2012). Compensatory enlargement of remaining synapses has been reported and may mitigate some of the early losses in spine density; as AD progresses, however, spine loss exceeds synaptic growth leading to a net reduction in synaptic transmission (Scheff et al., 1990). Further progression of AD results in loss of dendritic complexity (reduced length, less branching, changes in dendrite diameter) and eventually cell death (Alpár et al., 2006).

An important pathology associated with synaptic dysfunction is the impairment in the synaptic plasticity mechanisms hypothesized to underpin learning and memory. The most extensively studied form of synaptic plasticity, long-term potentiation (LTP), is reliably impaired in most animal models of AD and can also be caused by extracts obtained from post-mortem AD brain (Oddo et al., 2003; Shankar et al., 2008; Li et al., 2011). The impairment of LTP observed in animal models and from raised Aβ levels may in part relate to altered transmission and loss of dendritic spines (reviewed by Spires-Jones and Knafo, 2012), as well as impairments in N-methyl-D-aspartate (NMDA) receptor expression and inhibition of LTP-associated *de novo* protein synthesis (Snyder et al., 2005; Li et al., 2011).

The treatment of AD has proven to be extremely challenging. Despite an exhaustive array of clinical trials that now number in the hundreds (Schneider et al., 2014), no disease-modifying treatments have proven effective for clinical use, although there is renewed hope arising from a recent study that has given very promising results from anti-Aβ antibody treatment (Sevigny et al., 2016). On the other hand, a lack of significant cognitive improvements was recently reported for a phase III clinical trial in patients with mild AD (EXPEDITION-3) using Solanezumab, an anti-Aβ antibody that binds only soluble Aβ (Hawkes, 2016,^1^). Thus, at present only two classes of drugs have been approved by the Food and Drug Administration for AD treatment and these only address the symptoms of the disease (Geldenhuys and Darvesh, 2015). Acetylcholinesterase inhibitors (e.g., donepezil) target the reduced cholinergic innervation of the hippocampus and cortex resulting from the loss of basal forebrain cholinergic neurons (Whitehouse et al., 1982), and memantine targets the increased tonic activation of extrasynaptic NMDA receptors that leads to activation of apoptotic pathways and neuronal death (Hardingham and Bading, 2010). While these treatments provide some symptomatic relief, their efficacy invariably reduces over time and ultimately they fail to halt or reverse the progression of the disease. Therefore, it is vital that new treatment options continue to be explored.

## A Shift in the Balance of α-Secretase Versus β-Secretase Activity?

The amyloid cascade hypothesis has been the most widely supported explanation of the pathology that drives the progression of AD (De Strooper and Karran, 2016; Selkoe and Hardy, 2016), although other elements of the neuropathology are gaining increasing attention (Herrup, 2015; Rius-Pérez et al., 2015; Briggs et al., 2016). The amyloid cascade hypothesis contends that there is either a shift in APP processing towards the amyloidogenic pathway, or there is a reduction in Aβ clearance which results in the excessive accumulation of Aβ and a shift in the ratio of the various Aβ species to favor Aβ42. There is also evidence that BACE1 is upregulated during aging and AD, thus favoring amyloidogenic APP processing (Fukumoto et al., 2002, 2004; Holsinger et al., 2002; Yang et al., 2003; Li et al., 2004; Ahmed et al., 2010).

With the firm focus on increased levels of both soluble and insoluble Aβ in the brain and cerebrospinal fluid (CSF) in AD, relatively little attention has been given to a possible associated reduction in α-secretase activity and thus a shift away from the production of APPsα that might amplify the toxic effects of Aβ, hyperphosphorylated tau and other neuropathologies. However, the evidence for a reduction in APPsα levels in AD is mixed. Measuring mixed alpha and beta forms of secreted APP, Kibbey et al. (1993) reported that levels of APPs in the CSF of AD patients were 3.5 times lower than that in healthy controls. Subsequent studies specifically measuring APPsα in CSF supported this finding (Lannfelt et al., 1995; Almkvist et al., 1997; Sennvik et al., 2000), and positive correlations between reduced APPsα levels and diminished performance in cognitive testing in both AD patients (Almkvist et al., 1997) and normal aged rats (Anderson et al., 1999) have been reported. The loss of cholinergic innervation from the basal forebrain to the cortex and hippocampus in the very earliest stages of AD may underlie the loss of APPsα production and this may be the driver for the shift to amyloidogenic processing of APP (Obregon et al., 2012).

On the other hand, there is also evidence that APPsα levels may not be changed in the early stages of sporadic AD (Perneczky et al., 2014). Several studies using newly developed methodologies have reported that APPsα CSF and blood plasma levels are unchanged in sporadic AD patients (Olsson et al., 2003; Perneczky et al., 2011, 2013; Rosén et al., 2012; Brinkmalm et al., 2013) with decreases only in advanced AD (Rosén et al., 2012) and in AD patients carrying the ApoE-ε4 allele (Olsson et al., 2003). One study has even reported an increase in APPsα levels in the CSF of AD patients (Rosén et al., 2012). Thus, a complete understanding of the pattern of APPsα production in AD and its significance will require more detailed study of AD patients and testing in animal models of the disease.

While the production of APPsα in the brain still needs to be fully understood, evidence from studies in humans and animals indicates that reduced APPsα levels can exacerbate AD symptoms. A mutation at the α-secretase cleavage site of human APP (APP770K687N) was found to cause early onset dementia. The mutation severely reduced α-cleavage and thus APPsα production, but at the same time led to the production of highly toxic Aβ species, hampering a clear interpretation of the specific impact of low APPsα levels (Kaden et al., 2012). However, Epis et al. (2010) demonstrated that hippocampal ADAM10/SAP97 levels (a complex required for synaptic ADAM10 localization) are reduced in AD patients, while activity-attenuating mutations in the prodomain of the human ADAM10 gene have been associated with AD (Kim et al., 2009; Suh et al., 2013). Reducing ADAM10 activity in adult mice by impairing its trafficking (Epis et al., 2010) or through forebrain-specific conditional ADAM10 KO (Prox et al., 2013) shifted APP processing towards Aβ production. Together these data suggest that reduced APPsα levels may contribute to the early stages of sporadic AD.

## Properties and Functions of APPsα

**Figure 2:**
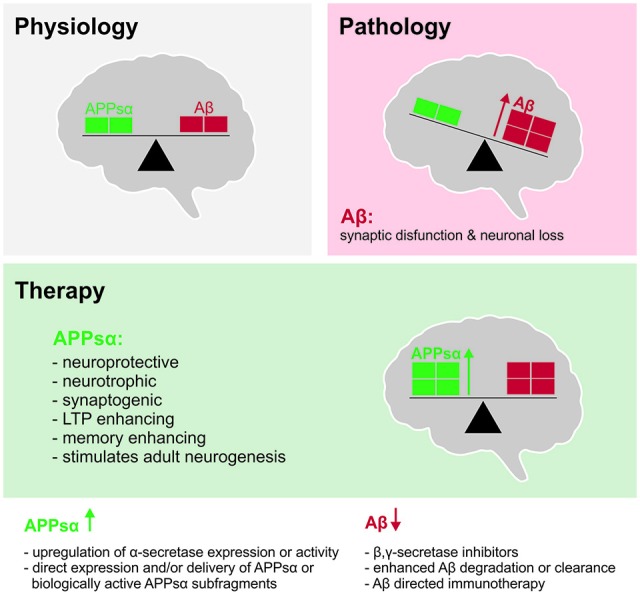
**Shifting the balance between APPsα and Aβ to ameliorate Alzheimer’s disease (AD). Top left** (gray): under physiological conditions, balanced levels of APPsα and Aβ are generated as products of normal brain metabolism. **Top right** (red): during AD, Aβ accumulates due to increased Aβ production and/or reduced Aβ clearance. Aβ accumulation in oligomers and plaques inhibits synaptic function, synaptic plasticity and cognition and ultimately leads to neuronal death. **Middle** (green): beneficial properties of APPsα that may be exploited to counteract the Aβ load and to ameliorate the symptoms and possibly the pathogenesis of AD. **Bottom**: strategies to shift the balance of APP processing towards the non-amyloidogenic pathway.

The possible significance of any impairments in ADAM10 activity or in the expression of APPsα becomes quickly apparent when one considers that this protein exerts a large number of growth factor-like properties when applied exogenously to neural tissue. Understanding the functionality of this protein, and its mechanisms of action, is crucial not only for understanding its biology in normal tissue, but also for providing critical information that will underpin any attempt to harness its potential therapeutic benefits (Figure 2).

## Neuroprotection

APPsα has strong neuroprotective properties that mitigate in cultured neurons the effects of a range of pro-apoptotic insults including hypoglycemia and glutamate toxicity (Mattson et al., 1993; Furukawa et al., 1996) and, importantly, Aβ-induced toxicity (Goodman and Mattson, 1994; Barger and Mattson, 1996a,b; Furukawa et al., 1996; Guo et al., 1998). More recently, we and others have demonstrated that the effects of other disease-associated insults such as excessive NMDA receptor activation (Ryan et al., 2013) and proteasomal impairments (Copanaki et al., 2010; Kundu et al., 2016) can be mitigated by APPsα administration. APPsα inhibits the upregulation of the co-chaperone BAG3 and suppresses BAG3-mediated aggresome formation under conditions of proteasomal stress (Kundu et al., 2016). Moreover, APPsα is a key activator of the PI3K/Akt survival signaling pathway that is triggered in response to serum withdrawal in cultured neurons (Milosch et al., 2014). Although the mechanisms conferring neuroprotection are only partially understood (for review see Kögel et al., 2012), some of these effects depend on the binding of APPsα to cell surface APP, that via its C-terminal domain can interact with G_0_ protein to trigger the pro-survival Akt kinase pathway (Milosch et al., 2014).

While most previous studies focused on cell death, the impact of APP on cellular and neuronal network functions during metabolic stress remain largely unknown. In this regard, Hefter et al. (2016) recently studied hypoxia-induced loss of function and recovery upon re-oxygenation in mouse hippocampal slices. While APP-KO mice showed impaired functional recovery after transient hypoxia, this could be largely rescued by APPsα expression or by pharmacological block of L-type calcium channels. Voltage-gated Ca^2+^ channels are, in addition to NMDARs and internal Ca^2+^ stores, major sources of intracellular calcium contributing to traumatic/ischemic insults and AD pathogenesis. These data indicated that APP, in particular APPsα, supports neuronal resistance against acute hypoxia by regulating calcium homeostasis (Hefter et al., 2016).

In addition to these *in vitro* studies, there is also evidence that APPsα may protect against acute forms of brain injury *in vivo*. Smith-Swintosky et al. (1994) demonstrated that APPsα ameliorates neuron loss in the hippocampus under conditions of transient ischemia, consistent with subsequent findings that APP-KO mice show increased acute mortality upon ischemia (Koike et al., 2012). In addition, a series of recent experiments have shown a protective effect of APPsα in traumatic brain injury (reviewed by Plummer et al., 2016). Intracerebroventricular (ICV) administration of APPsα following traumatic injury in rats significantly reduced cell and axonal death and improved motor outcomes (Corrigan et al., 2014). While APP-KO mice are more vulnerable to traumatic brain injury this could be rescued by recombinant APPsα or peptides derived from it (Corrigan et al., 2014; Plummer et al., 2016). Together these data indicate that endogenous APPsα is neuroprotective under injury conditions and suggest that these properties may be exploited in a therapeutic setting.

In a positive feedback cycle, APPsα may promote the further production of APPsα by blocking the amyloidogenic pathway through binding to and inhibiting the β-secretase BACE1 (Peters-Libeu et al., 2015), leading to a reduction in Aβ production (Obregon et al., 2012; but see also Fol et al., 2016). Further protection against AD-related toxicity by APPsα may arise from the inhibition of the tau phosphorylating enzyme GSK3β, thus reducing tau hyperphosphorylation and the subsequent production of NFTs (Deng et al., 2015).

## Trophic Functions: Cell Proliferation and Adult Neurogenesis

In addition to neuroprotection, APPsα exerts trophic functions both *in vitro* and *in vivo*. Early studies indicated that APPsα restores the growth of fibroblasts in which endogenous APP expression had been attenuated (Saitoh et al., 1989), stimulates thyroid epithelial cell growth (Pietrzik et al., 1998) and enhances the proliferation of rat fetal neural stem cells (Hayashi et al., 1994; Ohsawa et al., 1999). While these trophic functions appear beneficial under physiological conditions, enhanced APPsα expression has been detected in different tumors including glioblastoma (for review see Chasseigneaux and Allinquant, 2012). APPsα has also been implicated in adult neurogenesis. APP knockdown in adult mice resulted in reduced numbers of neurospheres that could be cultured form the ventricular zone (Caillé et al., 2004) and an APP-Fc fusion protein (Fc domain of IgG fused to the APP ectodomain) was shown to bind to the subventricular zone of adult mice (Caillé et al., 2004), suggesting that APPsα may stimulate neuronal stem/progenitor cell proliferation. Consistent with these findings, APPsα infusion into the lateral ventricle increased the number of EGF-responsive progenitor cells (Caillé et al., 2004), while pharmacological blockade of α-secretase by infusion of the inhibitor batimastat decreased the number of neuronal progenitors *in vivo* (Caillé et al., 2004). This was further corroborated by *in vitro* studies indicating that APPsα stimulates the proliferation of cultured neuronal precursor cells (NPCs) from the adult subventricular zone even in the absence of EGF (Demars et al., 2011) and also NPCs from the dentate gyrus (Baratchi et al., 2012). Consistent with the latter finding, transgenic overexpression of ADAM10 led to increased hippocampal neurogenesis (Suh et al., 2013). In addition, intraventricular injection of APPsα rescued the age-dependent decline in the number of NPCs *in vivo* (Demars et al., 2013). Taken together these findings indicate a prominent role of APPsα in adult neuronal progenitor cell proliferation.

## Role for Neurite Outgrowth, Synaptogenesis and Spine Density

Several *in vitro* studies indicated that APPsα can promote neurite (Clarris et al., 1994; Small et al., 1994) and axonal outgrowth (Young-Pearse et al., 2008). Several APP domains important for these functions have been identified including the N-terminal APP_96–110_ region located in the first heparin-binding domain and the APP_319–335_ region which contains the RERMS motif (Ninomiya et al., 1994). Studies from animal models also indicate a crucial role for APPsα in synaptogenesis and modulation of spine density. Using organotypic hippocampal cultures we have demonstrated a pronounced decrease in spine density and reductions in the number of mushroom spines thought to represent mature synapses in CA1 pyramidal neurons of APP-KO mice. Interestingly, APPsα expression alone was sufficient to prevent the defects in spine density observed in APP-KO mice, as APPsα knock-in mice that lack transmembrane APP and express solely the secreted APPsα fragment exhibited unaltered spine density and spine type distribution (Weyer et al., 2014). In line with this, APPsα could also partially restore spine density deficits of cultured APP-KO neurons (Tyan et al., 2012). In turn, these findings imply that autocrine or paracrine APPsα signaling, important for spine formation and/or maintenance, involves a so far unknown receptor distinct from APP itself. Further support for a synaptotrophic role of APPsα comes from transgenic mice with moderate overexpression of human wild-type APP (Mucke et al., 1996) or indirect up-regulation of APPsα by transgenic expression of the α-secretase ADAM10 (Bell et al., 2008), which both led to increased synaptic density. In Tg2576 mice, expression of mutant hAPP increased spine density in CA1 and cortical neurons of young mice prior to plaque deposition presumably via APPsα, whereas spine density was decreased in aged animals, likely due to Aβ-mediated synaptotoxic effects (Lee et al., 2010). This suggests that APPsα might counteract Aβ-mediated effects on spine density during early stages of pathogenesis. Recent evidence indicates that APP also regulates spine plasticity. Using two-photon *in vivo* microscopy, (Zou et al., 2016) analyzed cortical spine dynamics *in vivo* and reported decreased spine turnover rates (formation of new spines or loss of established spines) in APP-KO mice. Moreover, when housed under environmental enrichment, APP-KO mice failed to respond with an increase in spine density (Zou et al., 2016), suggesting that not only a reduction in spine numbers but also alterations in spine dynamics could contribute to deficits in synaptic plasticity and behavior found in APP mutant mice (Dawson et al., 1999; Seabrook et al., 1999; Ring et al., 2007). It remains to be seen which domains of APP or which proteolytic fragment is important for this function. The mechanism underlying the effects of APPsα on spines is presently unknown, although NMDARs could play a crucial role. APP-KO mice have decreased levels of extracellular D-serine (Zou et al., 2016), an essential endogenous co-factor of NMDAR activation (Panatier et al., 2006). Taken together these findings indicate important synaptogenic and synaptic modifying properties of APPsα that may be of therapeutic value (Fol et al., 2016; see also below).

## Synaptic Plasticity

Synaptic plasticity phenomena, such as LTP and long-term depression (LTD), are fundamental to learning and memory and are thus also central to normal cognitive function. In mouse models of AD, LTP is consistently impaired in an age-dependent fashion (Oddo et al., 2003; Vigot et al., 2006), and in some cases LTD is facilitated (Megill et al., 2015), while humans with diagnosed AD also show impaired synaptic plasticity (Trebbastoni et al., 2016). It is interesting to note then that APPsα has the capacity to facilitate LTP and thus has the potential to counter the LTP-impairing effects of Aβ. In an early study, Ishida et al. (1997) demonstrated that APPsα increased the frequency dependency of LTD induction in CA1 from 1 Hz to 10 Hz and facilitated LTP expression induced by 100 Hz stimulation, possibly by a protein kinase G (PKG)-dependent mechanism. Moreover, we showed in anesthetized rats that exogenously applied APPsα exerted an inverted U-shaped dose-dependent facilitation of LTP in the dentate gyrus, although too high a dose impaired LTP (Taylor et al., 2008). Moreover, APPsα antibodies as well as an α-secretase inhibitor impaired LTP, and the latter effect could be rescued by exogenous APPsα but not by APPsβ, despite its lacking only the 16 C-terminal residues when compared to APPsα (Figure 1B). The inhibition of LTP appeared to be mediated, at least in part, through a reduction of NMDAR currents generated during the high-frequency stimulation (HFS). No effects on basal AMPA or NMDA receptor currents were observed, suggesting that endogenous APPsα may be released during the HFS to contribute to LTP. However this point requires further study, as the effect of α-secretase inhibition on tetanic NMDA receptor currents was small, and other studies have reported both a decrease (Furukawa and Mattson, 1998) and an increase in single NMDA receptor currents (Moreno et al., 2015) in response to exogenous APPsα delivery. More recently, we generated conditional APP/APLP2 double KO (termed NexCre cDKO) mice that lack APP expression and thus APPsα secretion in excitatory forebrain neurons on a global APLP2-KO background (Hick et al., 2015). Consistent with findings by Taylor et al. (2008), this led to impairments in hippocampal LTP that were also reflected in impairments in hippocampus-dependent learning and memory tasks, including deficits in Morris water maze and radial maze performance (Hick et al., 2015). Interestingly, we demonstrated that acute treatment of brain slices with nanomolar amounts of recombinant APPsα, but not APPsβ, rescued the impairment of LTP (Hick et al., 2015). These findings indicate a crucial ability specifically for APPsα to support synaptic plasticity of mature hippocampal synapses on a rapid time scale. Similar differential effects of APPsα vs. APPsβ have been reported in assays of neuroprotection, with APPsβ being far less effective (reviewed by Chasseigneaux and Allinquant, 2012). Thus, the crucial functional domain of APPsα may reside within terminal APPsα-CT16 residues, and/or their presence alters the conformation of APPsα in a critical way. Indeed, there is evidence from recent structural analysis by small angle X-ray diffraction studies that the three-dimensional structure of APPsα is very different from APPsβ (Peters-Libeu et al., 2015). This study further suggested that the N-terminal E1 domains folds back towards the C-terminal juxtamembrane domain in APPsβ (Peters-Libeu et al., 2015). Thus, epitopes that are accessible in APPsα or when provided as peptides may become masked in APPsβ. This may have important functional implications as distinct 3D structures may enable or prevent binding to different receptors. Although the receptor(s) mediating the acute effects of APPsα on synaptic plasticity are currently unknown, they are not the endogenous APP and APLP2 that are both lacking in NexCre cDKO mice (Hick et al., 2015).

APPsα also appears to play an important role in processes of natural aging. Not only is memory performance correlated with APPsα levels (Anderson et al., 1999), but aging-related deficits in both LTP and cognitive behavior can be rescued by exogenous APPsα (Moreno et al., 2015; Xiong et al., 2016).

## Gene Expression and Protein Synthesis

Full expression of LTP requires gene expression and *de novo* protein synthesis, and this raises the question of whether APPsα itself directly regulates protein synthesis and the processes of translation and transcription that underlie it. Barger and Mattson (1996a) suggested that APPsα could regulate transcription through activation of the transcription factor NF-kappa B (NFκB), and extensive gene expression responses to relatively brief delivery of exogenous APPsα have been reported (Stein et al., 2004; Ryan et al., 2013). Gene expression responses occurred in as little as 15 min and these slowly changed from predominantly upregulation to predominantly downregulation during 24 h of APPsα treatment (Ryan et al., 2013). Upregulation occurred for immediate early gene transcription factors and for NFκB- and CREB-regulated genes, as well as regulation of late response genes known to be involved in cell survival, inflammatory responses, apoptosis and neurogenesis. These findings were further corroborated by Aydin et al. (2011).

Although APPsα can regulate coupled transcriptional and translational processes, it can also directly regulate protein synthesis. Claasen et al. (2009) found, using rat hippocampal synaptoneurosomes that are not transcriptionally competent, that APPsα initiated *de novo* protein synthesis in the dendritic compartment that was sensitive to the translation inhibitor cycloheximide. This effect was: (1) dose-dependent with higher concentrations failing to affect baseline protein synthesis; (2) age-dependent with a much reduced effect in tissue from aged rats; and (3) abolished by a PKG inhibitor and partially blocked by inhibitors of calcium/calmodulin protein kinase II (CaMKII), and mitogen-activated protein kinases (MAPKs). It appears likely therefore that at least part of the LTP facilitation by APPsα is through regulated transcriptional and translational processes, but this hypothesis has yet to be directly tested.

## Memory

Intracerebral administration of antibodies against the APPsα region of APP is able to cause learning and memory impairments in rat inhibitory avoidance (Doyle et al., 1990; Huber et al., 1993) as well as chick inhibitory avoidance (Mileusnic et al., 2000) tasks. Similarly, inhibition of α-secretase impaired rat spatial watermaze memory (Taylor et al., 2008) while APP knock-out impaired mouse spatial learning (Ring et al., 2007). Although these treatments are not specific manipulations of APPsα, it is notable that memory deficits could be prevented in a number of experiments by acute administration of either full-length APPsα (Taylor et al., 2008) or APPsα fragments (Mileusnic et al., 2000), or by genetic over-expression of full-length APPsα (Ring et al., 2007). APPsα and its fragments have also been used to rescue memory under other conditions of impairment, such as caused by the muscarinic receptor antagonist scopolamine (Meziane et al., 1998), Aβ (Mileusnic et al., 2004), head injury (Corrigan et al., 2012), and aging (Xiong et al., 2016). Moreover, viral vector mediated over-expression of APPsα rescued memory in a mouse model of AD (Fol et al., 2016).

There is also evidence that normal memory can be enhanced by exogenous APPsα or peptide fragments. Full-length APPsα enhanced go-no-go discrimination and operant lever pressing in rats (Meziane et al., 1998) while a 17-mer fragment (derived from the heparin binding domain located in the conserved E2 domain) facilitated spatial memory in the watermaze task for aged but non-memory impaired rats (Roch et al., 1994). A 5-mer peptide internal to that fragment converted short-term avoidance memory to long-term memory in chicks (Mileusnic et al., 2004). These findings need to be treated with caution, however, because transgenic over-expression of APPsα from gestation has been shown to lead to the development of autism-like markers such as hypoactivity and impaired sociability (Bailey et al., 2013), as well as aberrant T-lymphocyte development and function (Bailey et al., 2011).

## APPsα as a Therapeutic Target

The neurotrophic, neuroprotective, neurogenic, synaptogenic as well as neuronal plasticity and memory enhancing properties establish APPsα as an attractive therapeutic target during the early stages of AD and possibly also later. In this regard it should be kept in mind that due to the highly plastic nature of synapses, their dysfunction and loss are reversible processes. Thus, synaptic repair stimulated by trophic APPsα may ameliorate pathophysiology and improve clinical outcome as a complementary approach to eliminating toxic factors.

APPsα levels may either be enhanced by shifting APP processing towards the non-amyloidogenic pathway or by direct delivery/expression of exogenous APPsα (Figure 1). Inhibiting amyloidogenic APP processing, e.g., by targeting the Aβ-generating secretases has been a major focus of AD research over last two decades (e.g., Yan and Vassar, 2014; Geldenhuys and Darvesh, 2015) and several advanced BACE inhibitors are in phase 3 clinical trials (Cumming et al., 2012). However, using systematic proteomic approaches, it has become clear that all secretases have numerous substrates besides APP (Saftig and Lichtenthaler, 2015; Kuhn et al., 2016). Pharmacological inhibition of secretases may therefore have serious drawbacks due to mechanism-based side effects on other targets that are important for normal brain physiology. These concerns were further fueled by recent findings demonstrating that BACE inhibition upregulates non-canonical APP processing and production of Aη fragments that impair neuronal activity and LTP (Willem et al., 2015).

With respect to the alternative approach, enhancement of non-amyloidogenic APP processing may be achieved by upregulating α-secretase expression at the transcriptional level or by modulating its subcellular trafficking or activity (for review see Endres and Fahrenholz, 2012; Postina, 2012; Saftig and Lichtenthaler, 2015; Habib et al., 2016).

### Transcriptional Activation of ADAM10

The human ADAM10 promoter contains two retinoic acid response elements and ADAM expression can be upregulated at the transcriptional level by the vitamin A analog acitretin in cells and in transgenic AD mouse models leading to increased APPsα and reduced Aβ production (Tippmann et al., 2009). In a small clinical trial with AD patients, acitretin, that is already approved to treat psoriasis, was well tolerated and caused a significant increase in APPsα levels that was detectable in CSF samples of treated patients (Endres et al., 2014). Long-term studies with larger patient cohorts are planned. Melatonin, which decreases during aging and in AD patients, has been shown to efficiently decrease Aβ levels when administered at early stages of pathogenesis in Tg2576 AD mice (Matsubara et al., 2003). Recently, detailed *in vitro* studies indicated that the underlying mechanism involves plasma membrane-located melatonin receptor activation, and ERK1/2 phosphorylation leading to increased APPsα levels via transcriptional activation of ADAM10 and ADAM17 (Shukla et al., 2015). Moreover, and in line with data from Moreno et al. (2015) and Xiong et al. (2016), melatonin partially restored APPsα levels and spatial learning in aged mice (Mukda et al., 2016).

### Post-Transcriptional Activation of α-Secretase

Although the precise mechanisms of activation are not fully understood, it is clear that α-secretase activity, as judged by enhanced APPsα levels, can be directly or indirectly upregulated via ion channels, G-protein coupled receptors (GPCRs) and receptor tyrosine kinases. In particular, receptor-activated protein kinase C, MAP kinases, PI3 kinase and Ca^2+^ signaling have been shown to contribute to α-secretase activation. In many cases, however, it has not been directly studied which enzymes mediate increased APPsα production. In these cases processing may involve ADAM10 and/or ADAM17 and possibly further metalloproteases that have been shown to have APP cleaving activity *in vitro* (Saftig and Lichtenthaler, 2015). As a detailed description of these various pharmacological approaches is beyond the scope of this review, the reader is referred to a series of recent reviews (see Postina, 2012; Saftig and Lichtenthaler, 2015; Habib et al., 2016; Spilman et al., 2016).

Upregulation of α-secretase activity was reported for etazolate, an allosteric activator of GABA_A_ receptors, which increased APPsα in rat cortical neurons and guinea pig brain (Marcade et al., 2008), improved memory in aged rats (Drott et al., 2010) and proved protective against traumatic brain injury (Siopi et al., 2013). The neuropeptide pituitary adenylate cyclase-activating polypeptide (PACAP) potently increased APPsα levels, an effect that was abrogated by an antagonist of the GPCR PAC1, by a hydroxamate-based ADAM inhibitor and by inhibitors of MAP kinases and PI3 kinases (Kojro et al., 2006). *In vivo*, APPsα production in the brain was stimulated by long-term intranasal PACAP application. The effects of PACAP application were not limited to increased APPsα levels but were instead pleiotropic, including upregulation of the PAC1 receptor, BDNF and of the anti-apoptotic Bcl-2 protein (Rat et al., 2011). While these *in vivo* effects, including improved object recognition in transgenic AD model mice (Rat et al., 2011), appear favorable for treatment, Gardoni et al reported that PACAP treatment of primary hippocampal neurons led to postsynaptic ADAM10 accumulation and N-cadherin-dependent reductions in spine head volume and reduced postsynaptic GluR1 expression (Gardoni et al., 2012). Thus a more detailed *in vivo* characterization appears warranted.

Activation of serotonin type 4 receptors (5-HT_4_Rs), another class of neuronally expressed GPCR, promotes the activity of ADAM10 and APPsα generation. The 5-HT_4_R was shown to directly interact with the mature form of ADAM10 and agonist stimulation of the receptor accelerated ADAM10 activity by cAMP/Epac (cAMP-responsive Rap1 guanine nucleotide exchange factor) signaling (Cochet et al., 2013). Tesseur et al. (2013) reported that chronic 5-HT4 receptor activation lowered Aβ production in transgenic hAPP/PS1 AD model mice but the authors found no evidence for a direct activation of ADAM10. The underlying mechanism appears more complex and may involve decreased APP and BACE-1 expression and elevated astroglial and microglial responses. More recently, donecopride, a promising synthetic multitargeted ligand that functions both as a partial agonist of 5-HT_4_R and as an acetylcholinesterase inhibitor, has been developed and shown to have memory enhancing ability (Lecoutey et al., 2014).

### Direct Expression of APPsα in the CNS

Although α-secretase-targeting pharmacological strategies are potentially promising, there remains the concern regarding lack of specificity (see for example Gardoni et al., 2012). Acitretin may induce other genes with retinoid response elements in their promoters and upregulation of α-secretase activity (ADAM10, 17 or others) will likely lead to the processing of many additional substrates. In this regard, Kuhn et al. (2016) recently demonstrated that ADAM10 has over 40 neuronal substrates including some involved in tumorigenesis. Thus, it is still unclear whether increasing α-secretase activity in neural tissue will ultimately be of therapeutic benefit for patients. An approach to circumvent these problems is the direct delivery of APPsα into the brain.

While previous studies demonstrated the neuroprotective properties of APPsα against acute forms of brain injury (Van Den Heuvel et al., 1999; Thornton et al., 2006; Corrigan et al., 2012, 2014; Plummer et al., 2016) the situation is quite different in neurodegenerative diseases such as AD, characterized by chronic production and accumulation of neurotoxic molecules including Aβ. Another challenge is the need for sustained expression of neurotrophic/neuroprotective factors. This calls for a gene therapy approach. During recent years gene therapy approaches to neurological disorders including AD have been explored in preclinal studies and also entered phase I/II clinical trials (Tuszynski et al., 2015; Choudhury et al., 2016b; Fol et al., 2016; Hocquemiller et al., 2016). For the CNS, adeno-associated virus (AAV) vector systems have been most commonly used due to their safety, non-pathogenic nature, the ability to transduce dividing and non-dividing cells, particularly neurons *in vivo*, the wide volumetric distribution of vector particles in tissue and the ability to mediate long-term gene expression *in vivo* (Choudhury et al., 2016a; Hocquemiller et al., 2016).

Recently, we employed AAV9-mediated gene transfer of APPsα into the brain to explore its potential to ameliorate or rescue structural, electrophysiological and behavioral deficits of transgenic APP/PS1 AD model mice. A single bilateral injection of AAV-APPsα particles was sufficient to mediate long-lasting APPsα expression over 5 months that was well tolerated without apparent side effects. Interestingly, sustained APPsα overexpression in aged APP/PS1 mice with already preexisting pathology and amyloidosis restored LTP, ameliorated spine density deficits and also rescued spatial reference memory assessed by the Morris water maze. Moreover, we demonstrated a significant reduction of soluble Aβ species and plaque load. In addition, APPsα treatment induced the recruitment of microglia with a ramified morphology into the vicinity of plaques and upregulated IDE and TREM2 expression suggesting enhanced plaque clearance (Fol et al., 2016). These data further corroborate the therapeutic potential of APPsα for AD that raises hope to translate these findings into clinical application.

To this end further experimental studies including different routes of viral vector application, dose optimization and studies in lager animals are needed. Several routes of vector administration to the CNS have been developed: (i) direct injection into the brain parenchyma; (ii) CSF-based delivery using ICV, cisternal or lumbar intrathecal (IT) administration; and (iii) intravascular (e.g., intravenous) administration. Intracranial injection has been explored not only for diseases with anatomically restricted pathology such as Parkinson’s disease (reviewed in Choudhury et al., 2016b; Hocquemiller et al., 2016), but also for neuropathic lysosomal storage diseases (LSD) that affect large brain regions. For LSDs, multiple intraparenchymal injections were used in phase I/II clinical trials that showed the safety of the approach and also lead to encouraging clinical outcome (Leone et al., 2012; Tardieu et al., 2014). Vector delivery via the CSF’, in particular intracisternal and IT is a less invasive alternative strategy that is particularly promising for the delivery of secreted proteins such as growth factors and lysosomal proteins, and has been successfully used to express Apolipoprotein E in AD model mice (Hudry et al., 2013). Systemic, intravascular administration is the least invasive route and has the potential to enable wide spread vector distribution as every cell in the brain being a maximum of 40 microns from the microvasculature (Wong et al., 2013). In this regard encouraging progress has been made, as serotype AAV9 and AAVrh.10 have been shown to cross to some extent the blood brain barrier (BBB; reviewed in Hocquemiller et al., 2016; Saraiva et al., 2016), apparently by active transcytosis through endothelial cells (Merkel et al., 2017). The development of modified AAV vectors with re-engineered capsids should improve this further (Choudhury et al., 2016a; Deverman et al., 2016; Jackson et al., 2016). One of the main challenges for AD gene therapy is the widespread pathology that affects several anatomic regions involved in learning and memory. Thus, protocols that either target regions affected early during disease and/or widespread gene delivery to several anatomical regions are required.

A non-invasive alternative to viral vector mediated gene transfer are formulations of recombinant APPsα protein, sub-domains or active peptides that enable transport across the BBB. This includes intranasal delivery that has been successfully used in preclinical models of CNS diseases (Lochhead and Thorne, 2012). Examples for AD are the intranasal delivery of insulin (Mao et al., 2016) or PACAP (Rat et al., 2011) to enhance non-amyloidogenic APP processing in transgenic mouse models. Liposomes and nano-particle based approaches are emerging as further options (Kreuter, 2014; Khalin et al., 2016). Finally, transient opening of the BBB by transcranial focused or scanning ultrasound in combination with microbubbles might be used to further enhance delivery of viral vectors, proteins such as APPsα (or active fragments) and nano-particles (Thévenot et al., 2012; Leinenga et al., 2016).

Collectively, these various approaches all appear to merit further investigation. However it needs to be kept in mind that many challenges lie ahead for translating these approaches to the human brain, especially given its size and thus the widespread volume of brain tissue that needs to be treated. Moreover, the preclinical animal models being currently used do not fully recapitulate the human disease features, and thus successes in animal models need to be treated with caution. Nonetheless, despite these challenges the neuroprotective and synaptic repair inducing properties of APPsα make it a worthy target for future research aiming to treat AD, as well holding other neurological disorders.

## Author Contributions

BGM, MR, WCA and UCM co-wrote this review. MR designed the figures.

## Conflict of Interest Statement

The authors declare that the research was conducted in the absence of any commercial or financial relationships that could be construed as a potential conflict of interest.

## Footnotes

^1^http://www.ctad-alzheimer.com/live-webcast
